# How much do rheumatologists and orthopaedists doctors’ modalities impact the cost of arthritis in Cyprus?

**DOI:** 10.1186/s12891-015-0643-x

**Published:** 2015-08-14

**Authors:** Despena Andrioti, Kypros Kyprianou, George Charalambous

**Affiliations:** Centre of Maritime Health and Society, University of Southern Denmark, Niels Bohrs vej 9, 6700 Esbjerg, Denmark; Fredrick University, 7, Y. Frederickou Str., Pallouriotisa, Nicosia 1036 Cyprus

## Abstract

**Background:**

Osteoarthritis is one of the primary causes of long-term functional disability. With an estimated 13.5 % prevalence in the general population contributes to a significant financial burden both for patients and healthcare systems. The purpose of this research is to highlight the direct annual cost of the disease to the private healthcare sector of Nicosia.

**Methods:**

A questionnaire based on Greek and international research was completed between 10/1/2012 and 11/30/2012, with a sample of 20 doctors specialists in orthopaedics and rheumatology (50 % of practising physicians in the private sector). An assessment of the annual cost of medical procedures and tests, pharmacologic therapies (modalities) and supplies per patient followed, based on current costs. Direct costs were assessed through the micro-costing “bottom-up” approach. We isolated and separately priced the original diagnosis, followed by each stage of the disease.

**Results:**

The cost for the six predominant medical tests to establish a diagnosis and exclude mainly RA such as ESR, CPR, and X-ray as well as a physician’s office visit was 150€ per patient. The average direct cost per patient during stages 1, 2 and 3 of the disease was 280.54€, 1,834.64€ and 5,641.72€ annually, respectively, with an annual average of 2,573€ per patient.

**Conclusions:**

Even though during the period of the study, the country had not yet established clinical guidelines, the participating physicians followed international practices. Significant rise in the cost in each stage of the disease was found, with additional increases in the following years as a result of the expected increased prevalence of the disease. It is noted here that uninsured patients, as well as those who qualified for free medical care, they seek these services in the private sector, and had to pay out of pocket money for examination and treatment. These patients, thus, contended with a serious financial burden. Therefore, it is important to inform them very extensively regarding evidence-based management of the disease to aid them in coping with this chronic illness.

## Background

Osteoarthritis is a chronic illness, which presents as a result of degenerative joint disease primarily of the upper and lower extremities. It mainly affects the elderly population, but may in some cases affect younger generations. The main symptoms of osteoarthritis are joint pain, which intensifies as a result of movement and decreases when the patient is at rest [1, 2].

Osteoarthritis is a common disease and attributable to 50 % of musculoskeletal conditions. It is affecting 9.6 % of men and 18 % of women over the age of 60 internationally. It is notable that in 1990, it was estimated to be the 8th cause of disability internationally. With the increased life expectancy and aging of the population osteoarthritis will eventually be the 4th major cause of disability by the year 2020 [3]. The knee is the main joint affected by osteoarthritis, followed by the hip. Osteoarthritis of these joints affects approximately 10-12 % of the population over 65 years of age [4]. Under the age of 50, however, osteoarthritis is more prevalent in men, whereas, over the age of 50, it is more prevalent in women. This gender distinction continues and increases with age [5].

As research continues, it becomes more apparent that the onset of the disease is not brought on by a single cause. It appears that heredity plays a role in the development of osteoarthritis. In addition, extrinsic factors contribute in its development in persons with a genetic predisposition. The major causes for the disease seem to be age, obesity, occupational exposure (persons with occupational fatigue of the knee or hip joints; standing, repeated bending of the knee and hip joint and extensive walking), intensive practice in athletics, such as football, high jump, weight lifting, etc., previous injuries and anatomical irregularities [6]. About 80 % of those with osteoarthritis experience low quality of life as a result of feeling tenderness (pain) and restriction of movement [7, 8], whereas 25 % cannot function normally in their daily lives [9, 10].

To establish a diagnosis, physicians utilize the patient’s history and clinical examination. Therapy aims at alleviating the pain, preserving joint movement, as well as delaying further deterioration of the joint cartilage [11]. In order to achieve these objectives, pharmacological and non-pharmacological therapies are used [12]. Pharmacological therapy is related to the level of deterioration of the cartilage. Initially, a regiment of analgesics and anti-inflammatory medications could be prescribed. However, at later stages and in more serious cases, specialists may prescribe a range of modalities that are not always recommended for management of osteoarthritis by the professional bodies [13, 14] such as glucosamine or injections of hyaluronic acid administered directly into the joint in conjunction with strong opioids [15, 16]. Among the non-pharmacological therapies a significant role play weight loss, physical therapy, exercise, occupational therapy, patient education, as well as support from their social networks [7, 17–20].

Even though current guidelines for the management of osteoarthritis of the hip and knee include recommendations for effective financial interventions in terms of treatment and disease management [18, 21, 22], osteoarthritis is an expensive disease. As a result of its high prevalence, it burdens both the health care systems and the patients [23–25]. Studies showed that the cost of arthritis represents 1.5-2.4 % of the health budget or around 1.2 % of the GDP in developed countries such as Australia, Canada, France, UK and USA [26–29]. To determine direct cost, pharmacological as well as non-pharmacological therapies must be taken under consideration. Whereas to determine indirect cost, other factors than those related to costs of medication are equally important including absenteeism, reduced productivity due to pain, sick benefits, informal care cost, and premature mortality, depending on which agent’s (employer, patient, insurance company, society) perspective we examine. Inconsistencies in methodology make it difficult to collect accurate cost data, and thus comparisons in between countries and healthcare systems are weak.

The research on the cost of osteoarthritis is very limited in Cyprus. Therefore, our aim was to capture the direct annual cost for each patient diagnosed with osteoarthritis of the lower extremities, managed by a private orthopaedist or rheumatologist at the Province of Nicosia, with regards each one of the three stages of the disease. We considered annual cost in the private sector more representative, as it represents the market prices. It is totally paid by the patient as out-of-pocket money.

Further, we wanted to highlight the prevalence of the problem of osteoarthritis compared to other diseases treated by these private practitioners as well as to show the applied methods of management and treatment of osteoarthritis by these physicians.

Cyprus with a population of about 800.000 lies in the outermost borders of the European Union. In the country two parallel health systems operate, a public and a private one. The public system covers 85 % of the population while the rest has to pay out of pocket money in order to get health services. The out-of-pocket payments account for about 40 % and it is one of the highest proportions of household spending in Europe. The system is fragmented and the coordination and communication within and between public and private sectors is poor. It is very common even patients who are entitled for free medical care in the public sector to seek these services in the private, mainly due to long waiting lists in the former [56].

## Methods

### Doctors’ population

Initially, the number of rheumatology and orthopaedic specialists who were working in the private sector in the Province of Nicosia was recorded, amounting to 40 physicians (1:1000 residents). These physicians were personally contacted. A detailed explanation was given as to the aim of the study, as well as information on the potential benefits they could gain from a possible participation in the study, meaning that participation would enable them to communicate their diagnostic and therapeutic modalities for the treatment and management of patients with chronic osteoarthritis. In addition, they would have the opportunity to compare their diagnostic methodologies to those of their colleagues. They were assured that patient confidentiality would be carefully safeguarded. The study sample was 20 doctors, of whom 15 were orthopaedists and five rheumatologists, which was 50 % of these private practice specialists of the Province of Nicosia.

### The tool

The questionnaire (appendix) was built in accordance with international bibliography [11, 14] and based on international guidelines. It included 25 questions relating to physicians’ profiles, the diagnostic and therapeutic methodologies they used during each stage of osteoarthritis, and finally, any preventative and health educational measures, which they recommended to their patients for management of the disease. The completion of the questionnaire took place between 10/1/2012 and 11/30/2012.

### Stages

The stages of the disease, as prescribed by clinical plus radiographic criteria according to the American College of Rheumatology [30–32], were as follows: Stage 1: mild symptoms, Stage 2: strong symptoms, Stage 3: stronger symptoms which may lead to immobility, Stage 4: severe symptoms, which may lead to surgery and which are not the subject of this study.

### Ethics

There was no breach in patient privacy. We did not use patients’ personal data. Therefore relevant consent was not needed. The study protocol was submitted to and approved by the national authorities i.e. the Cypriot National Committee of Bioethics, the Cypriot Data Protection Authority and the Ministry of Health. The medical doctors who participated in the study completed the questionnaire so that we considered that we have their consent.

### Estimation of direct cost

Our aim was to estimate the direct average annual cost per patient in the different stages of the diseases. Patient information was reported in a technical formal descriptive systematic way. Some doctors utilized the relevant data at their disposal while others used their experience to report information. We applied the process of micro-costing, which is known as "bottom-up approach" [33]. The initial physician’s office visit/diagnosis was priced separately, as well as the first three stages of osteoarthritis.

In order to estimate osteoarthritis patient’s direct annual cost during each stage of the disease, we recorded the fees for the medical tests required for the initial diagnosis and for follow up with proper treatment for each stage.

To evaluate direct annual costs, pharmacological and non-pharmacological and adjunct modalities were recorded. Fees as listed on the webpage of the Ministry of Health were used in order to price medical practices, examinations, tests, medications and supplies. The Association of Physical Therapists provided the established fees for physical therapy. Private diagnostic centers provided established fees for laboratory tests and X-Rays. And finally, the Medical Association provided established fees for physicians’ office visits and treatments. An Excel table was used for each doctor to establish the descriptive statistical analysis of the data and calculate the frequencies of the variables. We collected for every doctor their weekly, monthly and annual modalities per patient (pharmaceuticals, supplies and laboratory tests) and added the number of visits, for each one of the different stages of the disease. We then calculated the average charge per patient for these visits and modalities (pharmaceuticals, supplies and laboratory tests) and adjusted it per patient per year.

## Results and discussion

Among the physicians participating in the study, 92 % were males. The majority, (35 %) were 60+ years old.

The number of patients with osteoarthritis amounted to 8,550 (30 %) out of the total number of 28,500 patients that the participating physicians treated. Among those, 3,420 (40 %) were males, whereas 5,130 (60 %) were females. 6,412 (75 %) were Greek Cypriots, 1,282 (15 %) were citizens of other European Union member countries, and 855 (10 %) were Turkish Cypriots. Regarding medical insurance, 3,847 (45 %) were eligible for card A, government health insurance (public servants are eligible for this type of insurance as well as vulnerable groups of the population with incomes less than 15,000€), 940 (11 %) had private health insurance, 769 (9 %) were insured by various agencies, and the remaining patients 2,992 (35 %) were uninsured.

**Fig. 1:**
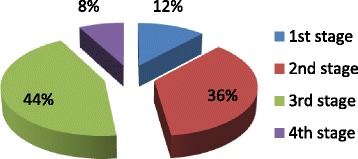
Breakdown of patients enrolled in the sample of physicians lists by stage of disease

With reference to the various stages of the disease, the participating physicians responded that they were treating 1,026 (12 %) patients who were in stage 1 of osteoarthritis, 3,078 (36 %) in stage 2, 3,762 (44 %) in stage 3. The remaining patients were in the last stage of the disease, in other words the stage prior to surgery (Fig. 1).

During the first stage of the disease, the participating physicians responded that their patients did not need sick leave. During the second stage, 308 (10 %) patients required 2–5 days of sick leave. During the third stage of the disease, 2,257 (60 %) patients required 2–5 days sick leave. Finally, during the fourth stage, 102 (15 %) required 7–10 days sick leave, whereas 581 (85 %) required 10 or more days of sick leave or even a certificate of disability.

The last question (appendix) addressed new incoming cases of osteoarthritis that the participating physicians treated each year, which were close to 25 per physician. Concerning patient referral to surgery within the past year, the participating physicians responded that 30 of their patients had surgeries of the hip and knee.

With reference to the comprehensive management of the disease, all participating physicians (100 %) provided preventive health care, counseling counselling on disease self-management, weight loss, exercise and nutrition.

## Cost of diagnosis

With an aim of diagnosing and placing the patient in the respective stage of disease (primary, secondary, or tertiary stage), all physicians prescribed X-rays of the affected joint (100 %), whereas 85 %-95 % of the physicians responded that they prescribed tests such as ESR, to classify patients with RA or OA. Furthermore, C-Reactive Protein (CRP) test was done to determine the level of inflammation. An MRI was prescribed with a frequency of 45 %, whereas a CAT scan was prescribed with a frequency of 65 %. Finally, synovial fluid analysis was prescribed with a frequency of 30 % and with a frequency of 20 % arthroscopy was used to positively diagnose the disease and its stages.

**Fig. 2:**
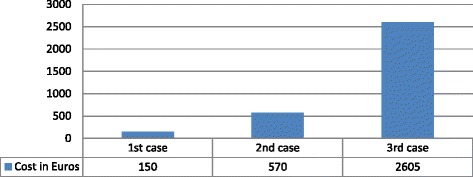
The average estimated cost to establish arthritis diagnosis in each of the three scenaria according to doctor’s modalities

Based on these data, we estimated the average cost for the diagnosis for three different scenaria of patients:

- First scenario: A patient would go through the most common used tests, such as ESR, CRP and X-ray used with a frequency of 85 % or more. The cost for such tests is estimated at 120€ per patient. The total diagnosis cost, including the physician’s office visit was estimated at 150€ per patient.
- Second scenario: A patient would go through tests, such as those used with a frequency of 45 % or more. In this case, the aforementioned tests would be administered, in addition to an MRI or CAT scan. The total diagnosis cost, including the physician’s office visit was estimated at 570€ per patient.
- Third scenario: A patient could go through all possible tests, such as those used with a frequency of 20 % or more. In this case, all the aforementioned tests would be administered as well as a synovial fluid analysis and/or an arthroscopy. The total cost in this case, including the physician’s office visit was estimated at 2,605€ per patient. Figure 2 demonstrates the average estimated cost to establish a diagnosis.

## Stage 1 cost of treatment

During stage 1 the average number of visits to the private practitioner’s office was estimated at 1.4 per patient per year. In addition, the total number of participating physicians responded that they prescribe an X-ray annually.

The pharmacological treatment during stage 1 was limited to simple analgesics and topical gels. The participating physicians prescribed paracetamol pain medications. Recommended dosage did not exceed one box per month. The average per patient monthly cost for these medications was 4.41€ monthly or 52.86€ annually.

In addition, they prescribed topical gels, such as mucopolysaccharide, polysulphate and salicylic acid, diclofenac and levomenthol. Recommended dosage did not exceed one prescription per month with an average cost of 6.02€ monthly or 72.18€ per patient annually.

Moreover, 65 % of doctors prescribed non-pharmacological (adjunct therapies), such as special shoes (anatomic) with an average cost of 60€ per patient, and (35 % of them) prescribed special stockings/socks with an average cost of 45€, two pairs annually. Table 1 depicts the average cost per patient with 1st stage osteoarthritis annually, which exceeds 280.54€ annualy.

**Table 1:** Annual average cost per patient with stage 1 osteoarthritis

Type	Cost €
Visit	42.00
X-Ray	40.00
Pain medications	52.86
Gels	72.18
Shoes and socks	73.50
Total	280.54

## Stage 2 cost of treatment

During stage 2, the average physician’s office visits was estimated at 1.7 per patient with an average cost of 51€. An X-ray was prescribed annually, similar to stage 1.

The participating specialists prescribed paracetamol and paracetamol/opioid pain medications at this stage. Recommended dosage did not exceed one box per month. The average cost was 5.84€ monthly or 70.03€ per patient annually. In addition, they prescribed the same topical gels, such as mucopolysaccharide, polysulphate and salicylic acid, diclofenac and levomenthol. Recommended dosage was 1.55 per month with an average cost of 11.88€ monthly or 220.97€ per patient annually.

During stage 2, anti-inflammatory medications, such as etoricoxib and diclofenac were prescribed with an average of 1.25 boxes per month, at a cost of 18.48€ monthly or 221.73€ per patient annually. Furthermore, gastroprofylaxis medicines were prescribed for all patients, such as pantoprazole and esomeprazole. Recommended dose did not exceed one box per month, with an average cost of these medications of 16.15€ monthly, or 193.76€ per patient annually.

Moreover, 30 % of the sample were prescribed dietary supplements, such as collagen with an average cost of 17.40€ monthly or 208.80€ per patient annually.

In addition, the participating physicians prescribed 2.15 physical therapy sessions with an average cost of 64.50€ monthly or 774.00 per patient annually.

The participating physicians (65 %) also prescribed nonpharmacological therapies such as orthopedic shoe inserts with an average cost of 250.00€ per patient, special shoes (anatomic) with an average cost of 60€ per patient, and (35 %) prescribed special stockings/socks (two pairs annually) for osteoarthritis with a total cost of 90.00€ per patient. The total average cost of these means was 132.76€ per patient annually. Table 2 depicts the average cost per patient with stage 2 osteoarthritis which exceeds 1,837.64€ per patient annually.

**Table 2:** Annual average cost per patient with stage 2 osteoarthritis

Type	Cost €
Visit	51.00
X-Ray	40.00
Pain medications	70.03
Anti-inflammatory	221.73
gels	142.56
Gastro prophylaxis medicines	193.76
Dietary supplements (collagen)	208.80
Physiotherapies	774.00
Adjunct means (shoes, socks, insoles)	132.76
Total	1,837.64

## Stage 3 cost of treatment

During stage 3 the average physician’s office visits were estimated at 5.35 per patient with an average annual cost of 160.50€ per patient. It appears that 40 % of the participating physicians prescribed an annual synovial fluid analysis with an average cost of 14€, an annual X-ray and an MRI (55 %) with an average cost of 205€ per patient.

The participating physicians prescribed parecetamol/opioid pain medications similar to stage 2. Recommended dosage did not exceed two boxes per month. The average cost was 12.38€ monthly or 148.50€ per patient annually.

In addition, the same topical gels were prescribed as in stage 2. The average use of topical gels was reduced in comparison to the second stage and amounted to 220.97€ per patient annually.

The same anti-inflammatory medications were used during this stage as in stage 2 of the disease with an average prescription of 1.45 box per month, with an average cost of 33.93€ monthly or 407.15€ per patient annually.

Furthermore, gastroprofylaxis medicines were prescribed as in stage 2 of the disease. Recommended dose did not exceed two boxes per month. The average cost of these medications was 46.87€ monthly or 562.41€ per patient annually.

In addition, an injection of hyaluronic acid was recommended by 70 % of the participating physicians, to be administered annually. The average cost for this treatment was 280.00€ per patient. 60 % of the participating physicians also recommended annual corticosteroid injections. The average cost for this treatment was 42.00€ per patient annually.

Moreover, 40 % of the participating physicians prescribed dietary supplements, such as collagen with an average cost of 23.20€ monthly or 278.40€ per patient annually.

Physical therapy was prescribed on an average 8.65 times per month, with an average cost of 259.50€ monthly or 3,114.00€ per patient annually.

The participating physicians considered certain supportive means for assistance necessary at this stage. As a result, canes and special stockings for osteoarthritis were recommended at a cost of 40€ and 80€ per patient respectively. About 60 % of the participating physicians recommended orthopaedic shoe inserts with an average cost of 250.00€ per patient, whereas special shoes (anatomic) with an average cost of 60€ per patient were recommended by 40 % of the sample. The total average cost of these means was 304€ per patient annually. Table 3 depicts treatment cost during stage 3 of the disease, which exceeds 5,641.72€ per patient annually.

**Table 3:** Annual average cost per patient with stage 3 osteoarthritis

Type	Cost €
Visit	160.50
Synovial fluid analysis	14.00
X-Ray MRI	205.00
Pain medications	148.5
Anti-inflammatory	407.15
Gels	125.76
Gastro prophylaxis medicines	562.41
Dietary supplements (collagen)	278.40
Corticosteroid injections	42.00
Hyaluronic acid	280.00
Physiotherapies	3.114.00
Adjunct means (shoes, socks, insoles)	304.00
Total	5,641.72

**Fig. 3:**
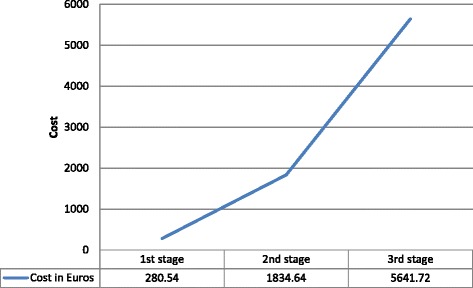
Patient’s average annual cost of osteoarthritis during each of the three stage

In Fig. 3, the average cost per patient annually appears.

## Discussion

Osteoarthritis is a chronic illness that affects mainly middle-aged and older adults, causing mobility problems and diminishing their quality of life [34]. As a result of the generally increased life expectancy, patients with chronic osteoarthritis are also expected to live longer, but with their deteriorating health, they are subjected to a constant increase in treatment costs posing a substantial burden to national health systems, insurance funds and family budgets [6, 35–37].

Based on outcomes from the present study, it appears that osteoarthritis is a very serious healthcare issue in the country, since 30 % of patients over 40 who visit the 20 participating specialists suffer from osteoarthritis. In addition, in Cyprus there is a free access to medical specialists, in other words, there is no gate keeping system. As a result, the patients visit orthopedists and rheumatologist as a first choice practitioner for diagnosis and treatment and mostly in the private sector [38, 39]. More specifically, similar findings from other studies showed that women, well-educated individuals and people with worse health status are more likely to visit private practice providers.

As expected, osteoarthritis affects females (60 %) more than males. The fact that most patients who visited the doctor were in stages 2 or 3 of the disease, points not only to the importance of preventive care, but also indicates a need of empowering patients to manage their illness. It appears then that patients in stage 1 of the disease as a result of mild symptoms do not have substantial difficulty in their daily lives. In addition, as expected, patients in stages 2 and 3 of the disease require more sick days. Regarding to the development of the disease, the private orthopedists and rheumatologists in Nicosia treat an average of 25 new patients each annually.

All participating physicians made recommendations for better disease management, exercise, weight loss, physical therapy and following a better nutrition regiment.

Alternative therapies for osteoarthritis, as suggested by the current bibliography [40], such as acupuncture, Tai Chi, Oigong and Yoga are not commonly used, nor recommended by the participating physicians in our country.

The study also showed that participating physicians followed scientific developments in their specialties and were well informed about diagnostic [15] and therapeutic recommendations [41] as appears from their management, which was similar with very few exceptions. Private practice specialists should take into consideration the cost of recommended modalities together with their efficiency, and this is very important in light of the current financial situation in the country.

As shown by this study specialists in Cyprus offer more services to osteoarthritis patients [42–45]. Laboratory tests prescribed for the diagnosis and placement of the patients in the correct stage of the disease as described by the private practitioner such as ESR, CRP and RA together with X-ray were primarily six, which in conjunct with the cost of the physician’s office visit were estimated at 150€ per patient. If an MRI or CAT scan were additionally prescribed the cost exceeded 570€ per patient. Finally, if patients were subjected to a synovial fluid analysis and/or arthroscopy, the cost for diagnostics would exceed on the average 2,600€ per patient.

The cost to patients in stage 1 of osteoarthritis exceeded on average 280.54€ annually and included analgesic medications, such as paracetamol pain medications and topical gels, such as mucopolysaccharide, polysulphate and salicylic acid, diclofenac and levomenthol.

During stage 2 of the disease, the cost for analgesic medications (paracetamol /opioid) and topical gels increased, because they were ingested more frequently. In addition, according to the majority of the participating physicians, during stage 2, patients were prescribed anti-inflammatory medications in conjunction with PPI’s for gastroprophlaxis that were from the other medications prescribed, as well as nutrition supplements. The most important increase in cost, however, was brought on by the use of nonpharmacological treatments, as well as physical therapy, which contribute in increasing the average direct cost to 1,834.64€ per patient annually.

Compared to the prior stages of the disease, the cost climbed exponentially during stage 3. The patients had to visit the physician’s office more often and many needed assistance via artificial means to walk. In addition, during stage 3, physical therapy and medications were prescribed more frequently consequent to pain increase. The extensive deterioration of the joint led participating physicians to use corticosteroid or hyaluronic acid injections annually, which aided in strengthening the elasticity of the joint. In addition, during this stage, patients were subjected to additional tests so that their physicians had a clearer picture of the patients’ need for surgical intervention. As a result, the patient with stage 3 osteoarthritis dealt with additional costs from the added synovial fluid analysis and MRI. The average direct cost exceeded 5,600€ per patient annually.

Overall, the cost for the disease has increased during the last decades. On the average, one third of the direct expenditures for osteoarthritis goes to pharmacologic therapies, many of which are used to alleviate the pain caused by the illness [46, 47].

Our research showed that the average annual direct cost of osteoarthritis in Cyprus is substantial, and that it escalates as the disease progresses. It ranges between 280.54€ for stage 1 patient annually, 1,834.64€ for stage 2 patient annually, and 5,641.72€ for stage 3 patient annually, resulting in an average direct cost of 2,573€ per patient annually. Similar international research showed an average total cost per patient ranging from 1,326€ in Italy [48–50] to 4,564€ in USA [26], while the direct cost varies from 403€ for France [51] to 4,040€ for Canada [52, 53].

It is difficult to draw concrete conclusions in terms of the factors that affect cost in every country. These variations may be due to different methods of cost calculations and highlight the importance of the harmonization of relevant cost measurement methods.

However, aggregated data at the national level highlight the importance of the issue and the economic burden of OA on the national health budgets of developed countries. More specifically, in 2001 in Australia, it was noted that over 7.3 % of the general population suffered from osteoarthritis and that the average cost of treatment exceeded 2.4 % of the total healthcare expenditures [54]. A similar study conducted in the USA in 2003 discovered that 27 million persons suffered from osteoarthritis, in other words 12.1 % of the general population, and that the average cost of treatment reached 1.2 % of the GDP [27–29]. In France, more than 13 million persons were examined to determine whether they had the disease at a cost of 1.7 % of the total expenditure of their healthcare system in 2002 [55] while in Spain, this exceeded 4.7 million euro [49].

The cost for the management of osteoarthritis depends on the number and range of services and the cost of treatment (medical, pharmacological, diagnostic and adjunct). The price of these treatments differs from country to country, and depends largely on labor fees as well as healthcare policies and cost of healthcare services, based on the supply and demand that are applicable to each country. In addition, different methodologies and lack of standardization in processing cost make comparisons between countries difficult.

Healthcare services for OA in Cyprus appear to be slightly higher than in other countries but in the same magnitude. It may be attributed to a larger number of prescribed laboratory tests, non-pharmacological support, and physical therapy. Regarding the increase in cost the method of payment (fee for service) could be blamed, which encourages the increase in the number of services rendered and contributes to the increase in cost, as well as lack of national guidelines implementation, coupled with patient demand for more services (adjunct means, diagnostic tests and medications), which contribute in the private practitioners’ compliance with such demands so that they do not lose their patients (customers).

Even though half of these patients qualified for free medical care in the public sector, they, nonetheless, preferred to seek medical treatment in the private sector. This demand of private practice healthcare services in Cyprus can be found in other studies as well. It would be interesting to see how the economic crisis influenced the patient’s behavior.

## Conclusions

The prescription patterns of medical specialists contribute to higher consumption and cost of services both for the patients and the health system. In line of a further increase of OA cost, that is expected, as a result of the rapid increase of two major determinants, aging and obesity, the central government should re-examine policies toward patients with chronic illnesses, creating a national policy for osteoarthritis. Doctor’s should be encouraged to use guidelines with an aim at improving quality, efficiency and cost containment. Emphasizing on the implementation of clinical guidelines by medical doctors and empowering patients regarding disease management and rational use of health services are major elements they should invest in the coming years. The current financial crisis in the country can help the reorganization of health services in terms of cost effectiveness in cooperation with all stakeholders.

## Limitation of the study

This study is only limited to the direct annual cost per patient. For a comprehensive analysis of the burden of OA indirect cost should be included as well. Furthermore, for an overview of the financial burden of disease it would be interesting to see the lifetime average cost of the disease.

The study refers to the private sector orthopaedic and rheumatologist physicians’ modalities in the region of Nicosia. Even though the sample was small (50 % of practicing physicians), one may expect to a certain extent similar results in the rest of the four regions as well as in those countries where the relation doctor/patient is characterised by the same values and beliefs as pointed out above. Furthermore, due to the fact that we did not use patients’ personal data, we did not match patients and doctors. Cultural reasons may lead the same patients visit more doctors for diagnosis and treatment and thus bias the number of patients with OA in the country.

## Abbreviations

ACR: American College of Rheumatology
CDC: Centres for Disease control and prevention
CMSS: Centre of Maritime Health and Society
CRP: C-reactive protein
CT scan: Computerized tomography
ESR: Erythrocyte sedimentation rate
EULAR: European League Against Rheumatism
RA: Rheumatoid arthritis
NICE: National Health and Care Institute of Clinical Excellence
MRI: Magnetic resonance imaging
OARSI: Osteoarthritis Research Society International
RA: Rheumatoid arthritis
SDU: University of Southern Denmark
